# The prevention strategies of swine viruses related to xenotransplantation

**DOI:** 10.1186/s12985-023-02090-3

**Published:** 2023-06-13

**Authors:** Hongzhen Mao, Jinyang Li, Guangneng Liao, Mengyu Gao, Guang Yang, Ji Bao

**Affiliations:** 1 Institute of Clinical Pathology, Key Laboratory of Transplant Engineering and Immunology, West China Hospital, Sichuan University, Chengdu, 610041 China; 2 Experimental Animal Center, West China Hospital, Sichuan University, Chengdu, 610041 China; 3 Center of Infectious Diseases & Institute of Clinical Pathology, Key Laboratory of Transplant Engineering and Immunology, West China Hospital, Sichuan University, Chengdu, 610041 China

**Keywords:** Swine viral, Xenotransplantation, Prevention & control, Epidemiology, Vaccination, Transgenic and gene-edited pigs

## Abstract

Xenotransplantation is considered a solution for the shortage of organs, and pigs play an indispensable role as donors in xenotransplantation. The biosecurity of pigs, especially the zoonotic viruses carried by pigs, has attracted attention. This review introduces several viruses, including porcine endogenous retroviruses that are integrated into the pig genome in a DNA form, herpesviruses that have been proven to clearly affect recipient survival time in previous xenotransplant surgeries, the zoonotic hepatitis E virus, and the widely distributed porcine circoviruses. The detail virus information, such as structure, caused diseases, transmission pathways, and epidemiology was introduced in the current review. Diagnostic and control measures for these viruses, including detection sites and methods, vaccines, RNA interference, antiviral pigs, farm biosecurity, and drugs, are discussed. The challenges faced, including those posed by other viruses and newly emerged viruses, and the challenges brought by the modes of transmission of the viruses are also summarized.

## Background

Xenotransplantation refers to the transfer of living cells, tissues, or organs between different species and is considered an alternative solution to the shortage of organs in allotransplantation. Pigs, due to their physiological similarity to humans, short production cycle and growth rate, and low risk of zoonotic diseases, play an irreplaceable role as donors for xenotransplantation. With the success of the first pig heart transplantation experiment, xenotransplantation is becoming a reality, but the safety of viruses remains a challenge for humans. Porcine cytomegalovirus (PCMV) is considered one of the major causes of patient death [1].

This review selects several viruses that are attracting attention in xenotransplantation, including porcine endogenous retroviruses (PERVs), herpesviruses (including PCMV and porcine lymphotropic herpesvirus), hepatitis E virus (HEV), and porcine circoviruses (PCVs), which are considered to have zoonotic potential. These viruses are also mentioned in the American Society of Transplantation’s guidelines [2]. The review introduces their basic situation and epidemiology in China. In addition, this review provides a reference for the detection and control of these viruses and lists some of the challenges posed by viruses in xenotransplantation.

## Several viruses closely related to xenografting

### Porcine endogenous retroviruses (PERVs)

PERVs are a subgroup of γ-retroviruses in pigs that can convert RNA to DNA and integrate into the host genome. [3]. PERV is a single-stranded positive-stranded RNA virus. While the pathogenicity of PERVs is relatively weak, their presence raises concerns about their potential for interference with xenotransplantation [4]. PERVs are capable of infecting certain immortalized human cells in vitro, leading to productive infections [5]. However, the ability of PERVs to infect nonhuman primates in vivo is limited. Even in the case of nonhuman primates that are immunosuppressed, PERVs do not cause infection in vivo [6]. According to the guidelines from the U.S. Department of Health and Human Services, pigs for xenotransplantation should have their cells cocultured with indicator cells for at least 30 days to test for infection of indicator cells and to consult the Center for Biologics Evaluation and Research [7].

Despite its weak interspecies transmission capacity, PERV is integrated into the genome and primarily spreads vertically and is therefore not susceptible to interruption through weaning or cesarean delivery procedures, resulting in its extensive global distribution [4]. PERV-A and PERV-B, as DNA copies, integrate into the genome of all pigs, while PERV-C is present in the majority but not all pigs [5]. Recombinant viruses between PERV-A and PERV-C have been identified in some pigs, which exhibit high replication rates and have the potential to infect human cells [8].

Certain Chinese miniature pigs contain fewer PERV copies than other pig breeds, making them potential candidates as donors for xenotransplantation [3]. A study evaluated the presence of PERVs in Chinese miniature pigs, including Guizhou (GZ), Bama (BM), Wuzhishan (WZS), and Juema (JM) breeds. PERV env-A and env-B were detected in all individuals. The proportion of PERV-env C varied among breeds, with 17.6% in GZ, 64.3% in BM, 83.3% in WZS, and 53.3% in JM, and their median number of PERV copies was 12, 16, 14, and 16, respectively [9]. Additionally, in additional studies, PERV-env C was detected in 100% of Ningxiang pigs [10], with a noteworthy observation that 90.5% (19/21) of Ningxiang pigs possessed PERV-A/C recombinants [10].

### Herpesvirus

In the field of xenotransplantation, pigs serve as vital donors [11]. While certain viruses present in pigs may not result in severe illness, they must not be disregarded in the immunocompromised state of xenotransplantation. Among these viruses, herpesvirus holds a significant position. Herpesviruses are large DNA viruses, and the herpesvirus family comprises three subfamilies: alpha herpesvirus, beta herpesvirus, and gamma herpesvirus [12].

#### Pseudorabies virus (PRV)

In pigs, PRV, also referred to as SuHV-1, not only induces Aujeszky’s disease in both domestic and wild pigs but also has a broad host range, including sheep, dogs, cows, mink, and other mammals [13, 14]. PR infection in pigs manifests clinically with symptoms such as diarrhea, vomiting, and disorders of the nervous system. Piglets within two weeks of age are particularly susceptible, with high rates of morbidity and mortality. Growing pigs may also experience difficulty breathing and impaired growth, while breeding pigs may encounter reproductive disorders. These issues collectively result in significant losses for the pig farming industry [15, 16].

Swine that recover from PRV excrete large amounts of virus in saliva and nasal secretions, and perhaps in urine and feces, for up to two weeks. Virus can persist in the tonsils of carrier swine for at least several weeks [17]. Latent virus can persist in the CNS for many months2. PRV is spread by several mechanisms, including direct (physical) contact, indirect contact, droplets and aerosols [18].

#### Porcine cytomegalovirus (PCMV)

PCMV, also known as SuHV-2, belongs to the genus Roseolovirus. It has been officially named Suid betaherpesvirus 2 [1]. This virus is prevalent in pigs and can cause fetal or neonatal death in affected animals, as well as being associated with small body size, rhinitis, and pneumonia in piglets [19].

In the context of xenotransplantation, PCMV has been found to spread to nonhuman primates, resulting in a significant reduction in the survival time of xenotransplanted animals [20]. Previous studies have shown that the presence of PCMV during porcine kidney transplantation in baboons and crab-eating monkeys resulted in survival times decreasing from 53 days to 14 days and from 28 days to 9 days, respectively [21, 22]. In 2020, a study found that the in situ transplantation of pig hearts with active PCMV/PRV in baboons resulted in a reduction in survival time from 195 days to less than 30 days [23]. Furthermore, in 2022, the University of Maryland in Baltimore conducted the first xenotransplantation of a pig heart to a human patient, in which PCMV may have contributed to the death of the patient as it entered the patient’s body with the transplanted organ [1].

PCMV is a widespread pathogen found in pig populations across the globe [24], with subclinical infections being more common than clinical infections. Serological studies conducted in the UK have shown that over 90% of pig herds have been exposed to PCMV infection [25]. The virus can be shed through various bodily secretions, such as nasal and ocular discharges, urine, and farrowing fluids [25].

#### Porcine lymphotropic herpesvirus (PLHV)

Porcine lymphotropic herpesviruses 1, 2, and 3 (PLHV-1, PLHV-2, and PLHV-3), also known as SuHV-3, SuHV-4, and SuHV-5, are viruses of the gammaherpesvirus family [12]. These viruses are commonly found in both domestic and wild pig populations. Despite their high prevalence, they do not have a significant impact on the pig farming industry [26, 27]. While PLHVs may not cause disease in their natural host, they can be pathogenic in other species [28]. It is noteworthy that PLHVs have been associated with posttransplantation lymphoproliferative disorder (PTLD) in immunosuppressed swine that have undergone stem cell transplants [29].

porcine lymphotropic herpesviruses (PLHVs), primarily spreads horizontally, but vertical transmission is also possible [30]. In commercial pig populations in Italy, PLHV is widely present with a prevalence rate of 28.97% for PLHV-1, 10.79% for PLHV-2, and 4.54% for PLHV-3 [27].

### Hepatitis E virus

Hepatitis E virus (HEV) is a small nonenveloped virus with a diameter ranging between 27 and 34 nm and a single-stranded positive sense RNA genome [31]. At present, there are a minimum of eight distinct genotypes (gt) of HEV [32]. Among them, HEV gt3 and gt4 are of porcine origin and may infect humans, particularly in the context of immunosuppressed xenotransplantation. The majority of HEV gt3 and gt4 infections are asymptomatic, ranging from 67 to 98% of all infections; however, in isolated cases, these infections may result in mild jaundice or moderate hepatitis [33]. Consequently, efforts must be made to eliminate the presence of HEV in the pig donor during xenotransplantation [34]. HEV is mainly transmitted by fecal-oral transmission and contact. It is worth noting that even though the pig’s infection has ended, HEV RNA may persist in organs such as the liver. If humans consume pork or transplant pig organs, there may be a risk of infection [35].

HEV3 and HEV4 infect humans through contact transmission or consumption of contaminated food (such as undercooked meat) [36]. A 2014 survey report indicated that the seroprevalence of HEV in rural populations in China was 38%, with an incidence rate of 2.8/10,000 cases, with the majority of cases being identified as HEV-4 [37]. In recent studies, hepatitis E virus (HEV) RNA was detected in 6.3% and HEV IgG in 40% of 5,033 serum samples from market-weight pigs at 25 slaughterhouses in 10 US states[38]. The virus is present on most pig farms worldwide.

### Porcine circovirus (PCV)

Porcine circoviruses (PCVs) are characterized by single-stranded DNA genomes arranged in a circular configuration. The viruses have been sequentially designated PCV1, PCV2, PCV3, and PCV4 based on their discovery order. PCV2 has been identified as the primary causative agent of porcine circovirus diseases and associated illnesses [39]. Liu et al. reported that PCV2 is capable of infecting 12 human cell types in vitro [40]. PCV3 has a history of interspecies transmission in past heterograft transplantations [41], yet there is currently no empirical evidence of its ability to infect human cells in vitro.

PCV has a widespread presence in all tissues of pigs and the environment, such as contaminated water samples. This leads to contact transmission as well as interspecies transmission. PCV2 may even exhibit the potential for airborne transmission [42]. The clinical symptoms of PCV2 and PCV3 are similar, including respiratory system diseases, reproductive disorders, intestinal diseases, and porcine dermatitis and nephropathy syndrome (PDNS). Furthermore, PCV2 is capable of inducing postweaning multisystemic wasting syndrome (PMWS). Given these findings, it is concluded that PCV presents a threat to xenotransplantation.

PCV is highly prevalent worldwide and is responsible for enormous economic losses to pig producers. During the period of 2018 to 2020, a total of 198 samples collected from Central China were analyzed for PCV2 and PCV3. The results indicated that 113 (57.07%) and 72 (36.36%) samples were positive for PCV2 and PCV3, respectively, while 39 (19.7%) samples tested positive for a combined PCV2 and PCV3 infection [43].

## Strategies for detecting and controlling swine viruses

### The application of sensitive detection methods

In the context of xenotransplantation, a pressing issue is the identification of suitable donor pigs that are free of pathogens. Despite being raised under specific pathogen-free (SPF) conditions, it is possible for pigs to harbor viruses such as PERV or PCMV [4, 44]. As such, it is imperative to establish a sensitive diagnostic system for the screening of porcine pathogens. In the table below, the relevant detection methods and detection sites are summarized (Table 1).

#### Serological detection

Serological examination is often used to detect past infections. PCMV infection often establishes a lifelong infection, which can be detected by using immunological methods such as protein blotting and using recombinant fragments of viral glycoprotein B (gB) to detect PCMV-specific antibodies. Even the viral content in the pig during the incubation period may fall below the minimum threshold for detection by polymerase chain reaction (PCR) techniques [45].

#### Detection of nucleic acid based on PCR

For PCMV, the common PCR or real-time PCR method may result in false negatives. Thus, a more sensitive PCR method needs to be adopted and performed at an appropriate time. The improved duplex real-time PCR and nested PCR methods have a higher accuracy than commercial real-time PCR [46, 47]. For the time frame of detection, real-time PCR is easier to detect the presence of PCMV in piglets compared to antibody testing [1].

In addition to real-time PCR, droplet digital PCR (ddPCR) is also a means of virus detection. ddPCR is usually used for quantification of PERV viral copy number. Since the PERV copy number in different pig breeds is greatly different, quantifying the PERV copy number in different pig breeds using ddPCR is useful for screening the most suitable pig breed for xenotransplantation [9].

### Vaccination

Vaccination is one of the best means for preventing and controlling diseases caused by viruses. However, not all viruses have commercial or suitable vaccines. PRV has commercially available vaccines, such as Bartha-k61, which effectively combats the virus and its mutations [48]. In addition to Bartha-k61, PRV has many other types of vaccines, including inactivated vaccines, live gene-deleted vaccines, live attenuated recombinant vaccines, etc. [49], and vaccination is the most common method of preventing and controlling PRV.

Porcine Circovirus Type 2 (PCV2) has many commercially available vaccines, most of which are inactivated vaccines or subunit vaccines based on PCV2’s ORF2 protein [50]. However, these commercial vaccines cannot completely eliminate the virus, they can only prevent clinical symptoms by reducing the viral load, and pigs will still be infected with the virus [51]. Moreover, PCV2 ORF2 is only 31% similar to PCV3 ORF2, 45% similar to PCV4 ORF2, and 70% similar to PCV1 ORF2, making PCV2 vaccines less protective, especially emerging strains of other strains [52].

Currently, there is no commercial animal vaccine for hepatitis E virus (HEV). However, there are vaccine candidates available, such as the intramuscular vaccine derived from a gt 4 strain, HEV p179 [53, 54], and an oral vaccine with hepatitis E virus capsid protein and immunobiotic bacteria-like particles [53, 54] (Table 1).

### Application of RNA interference technology in porcine virus suppression

RNA interference (RNAi) is a quick and efficient way to silence gene expression. The process involves two steps: first, the dsRNA is broken down into small interfering RNAs (siRNAs) through RNase III-like activity; then, the siRNAs join with RISC (RNA-induced silencing complex) and degrade cognate mRNA [55]. When applied to viruses, the RNAi target is often the virus’s RNA (Table 1).

For example, HEV persists within cells and can lead to chronic infections and liver damage in individuals with weakened immune systems. Zhang et al. validated the three most efficient shRNAs targeting HEV GT3, which target the methyltransferase domain, the junction region between the open reading frames (ORFs), and the 3´ end of ORF2. After shRNA was transduced into cells, the replication of HEV in the system was reduced by as much as 95% [56]. In another report, shRNA designed to target the HEV ORF2 gene in HEV GT4 produced good protection in cells.

PERV-specific shRNA is utilized to reduce the release of infectious PERV particles both in vitro and in transgenic pigs [57, 58].

### Application of gene editing technology in porcine virus elimination

**Fig. 1:**
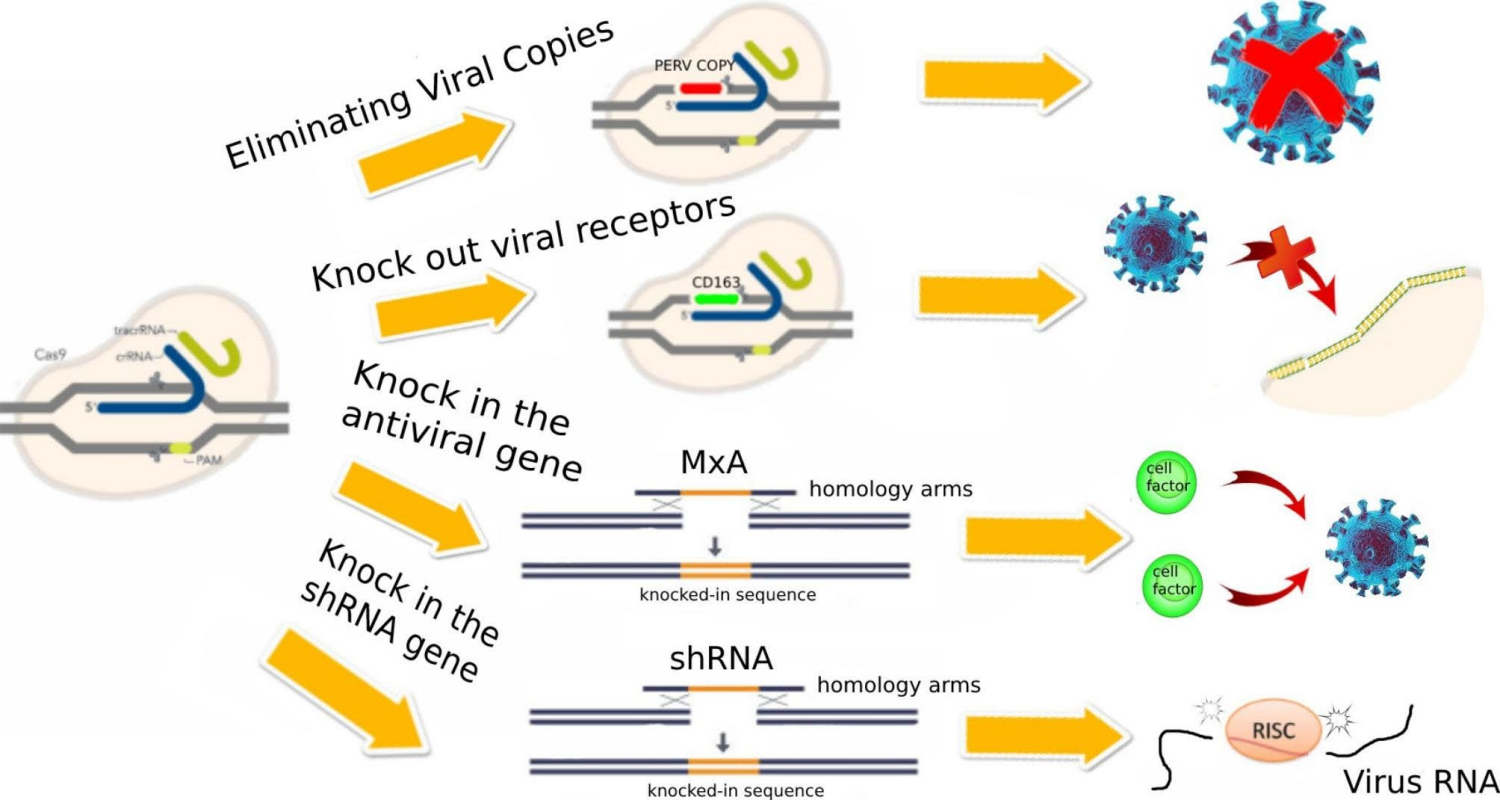
Gene Editing Technology in Porcine Virus Elimination

Genome editing technology, particularly that based on clustered regularly interspaced short palindromic repeats (CRISPR), can accurately modify the genetic material of pigs. This technology can directly eliminate viral copies or edit cell factors and receptors, thus avoiding virus infection (Fig. 1).

#### Gene editing technology directly eliminating viral copies

Most viruses can be eliminated by strict husbandry conditions; however, PERV can be introduced into the pig genome in the form of DNA, resulting in the inability to eliminate it through conventional means. However, with the application of CRISPR/Cas9, PERV can be eliminated through gene editing. Yang et al. completely eliminated 62 PERV copies in pig PK15 using CRISPR/Cas9 in 2015 [59], and in 2017, a PERV-free pig was cultivated [60]. In 2021, the team cultivated PERVKO·3KO·9TG pigs, which not only knocked out and knocked in genes related to xenotransplantation but also achieved complete inactivation of PERV [61].

#### Gene editing technology generates antiviral pigs by editing cell factors and receptors

Classic swine fever virus (CSFV) is a small, positive single-stranded, enveloped RNA virus, and the host genes MxA and pRSAD2 can play an antiviral role. In 2016, the Ouyang group cultivated pigs that overexpressed MxA [62], and in 2020, they cultivated pigs with specific integration of RSAD2 at the Rosa26 locus [63], both of which can inhibit the development of CSFV.

Gene knockout is also a means of resisting viral interference. Porcine reproductive and respiratory syndrome virus (PRRSV) is an enveloped, single positive-stranded RNA virus. PRRSV enters pig cells through various receptors, and CD163 is reported to be the key receptor in the PRRSV infection process [64]. In recent years, pigs with CD163 gene knockout using CRISPR/Cas9 technology have demonstrated resistance to PRRSV [65, 66].

#### The combination of RNA interference technology and gene editing technology

The effective and stable interference of RNA on viruses within pigs can be achieved through the combination of RNA interference technology and gene editing technology. This is achieved by integrating exogenous shRNA into the porcine genome through either a CRISPR/Cas9-mediated knock-in strategy or a transgenic strategy [67]. Transgenic pigs that express PRRSV-specific shRNA have been demonstrated to significantly inhibit the growth of PRRSV both in vitro and in vivo [68]. The expression of shRNA in porcine cells using a CRISPR/Cas9-mediated knock-in strategy has also been shown to produce similar results [67].

**Table 1:** Detection and removal of certain viruses

virus	PERV	PCMV	HEV	PCV
Source of the virus	Endogenous	exogenous	exogenous	exogenous
Detection method	Realtime-PCR, droplet digital PCR	Serological methods, duplex real-time PCR	Serological methods, real-time PCR	multiplex real-time PCR[69]
Detection sites	Any tissue, organ.	Serological testing performed on blood samples, nucleic acid testing performed on nasal swabs, spleen, kidney.	Nucleic acid testing performed on muscle, urine, serum, and feces samples.	In most tissues and body fluids, with serum and lymphoid tissue being the main sites
Virus removal from pig	Gene editing	PCMV can be removed with a high probability by early weaning, colostrum deprivation and cesarean derivation	Divide-column breeding, farm disinfection, isolate negative pig groups.	use commercial vaccines.
Genetically Modified Pigs	Gene-edited pigs with internal PERV copies partially or completely knocked out.	N/A	N/A	N/A
RNA interference	Anti-PERV shRNA targeting the gag and pol genes [57]	N/A	Targeting the ORF3 gene of HEV GT3.	Target sequence in the Rep gene encoding region [70]
vaccine	N/A	N/A	vaccine candidates, such as intramuscular vaccine derived from a gt 4 strain, HEV p179	Commercial vaccines, such as inactivated vaccines, or subunit vaccines based on the ORF2 protein of PCV2.

### Control of pig farms

#### Selection of pig herds

Not all pigs carry certain pathogens, and when introducing pigs into the pig farm, it is necessary to detect the pathogens carried by the pigs. In particular, viruses such as PERV and PCMV are not easily removed and carried for life. For example, low-expression PERV pig populations or pig populations that do not carry PERV-C can be selected for PERV.

#### Biosecurity of pig farms

Strict disinfection measures are necessary to effectively reduce the duration of virus attachment to objects such as pigs, humans, vehicles, and equipment. For example, for HEV and PCV, which can be transmitted through oral contact, biosecurity is essential. HEV may persist in drinking water and feces and in buildings [71–73]. Therefore, it is necessary to replace and disinfect the drinking water and feed of pig farms in a timely manner and to clean up the excrement produced by pig herds [74].

Second, improving the flow control measures of pig farms can help control the spread of the virus. Virus screening for new pigs entering the farm, separate management of piglets and fattening pigs, and reducing the mixing of pig herds are all measures that are conducive to controlling the spread of viruses. To prevent the entry of other pathogens and the coinfection of viruses, the replication of some viruses (e.g., PCV2) can be suppressed [39].

Some porcine viruses may be capable of vertical transmission; thus, it is necessary to interrupt transmission from sows to piglets. For example, PCMV can be prevented from vertical transmission through early weaning, colostrum deprivation, and cesarean Sects. [75, 76]. However, the prevention of PLHV transmission through cesarean section was only partially successful [77]. On the other hand, PCV2 is readily transmitted through the placenta, and colostrum has been proven to be infectious, making the elimination of PCV2 more challenging. Therefore, vertical transmission of PCV2 can be interrupted through cesarean delivery and the selection of colostrum from negative animal sources [78]. To achieve the ultimate elimination of this virus in pig herds, it is necessary to use highly sensitive detection methods to avoid false negative results on this basis; isolate virus-negative animals to prevent new infections, and use them as recipients for embryo transfer.

### Medicines

The appropriate drug can have a certain degree of control the virus and ameliorate the symptoms. But it’s worth noting that pigs infected with PERV and herpes viruses, even if they improve their symptoms after receiving medication, still carry the virus for life [46]. Medicines are often used as part of the regimen to prevent further transmission of the virus in pigs, but drug therapy is not an alternative to eliminate the virus. Therefore, virus-free negative pigs should be selected in the selection of donor or embryo transfer recipient pigs. The recent drugs used to control the virus are summarized in Table 2.

**Table 2:** Some medicines for certain viruses

virus	Medicine
PERV	Specific antiretroviral drugs that inhibit HIV-1 can also suppress PERV in vitro [79, 80].
PRV	There are a range of alternative compounds that are effective against PRV infection, such as resveratrol (trans-3, 4,5-trihydroxystilbene; Res) [81, 82], Kaempferol [83], Quercetin [84] etc.
PCMV	Cidofovir and foscarnet can inhibit PCMV in vitro [85].
HEV	pegylated interferon-alpha, ribavirin [36]
PCV	Some compounds are effective against PCV in vitro, such as Arctigenin (ACT) [86], but there is no systematic therapy.

## The challenges of porcine viruses in xenotransplantation

### Challenges from other viruses and emerging pathogens

On the one hand, there remain some viruses that pose a risk of causing zoonotic diseases and impacting xenotransplants. These viruses have been incorporated into the pretransplant screening regimen. However, new and potential viruses continue to threaten the safety of xenotransplants, as they have the potential to infect human cells or initiate another outbreak of porcine diseases.

#### Viruses with zoonotic potential in xenotransplantation: porcine rotavirus and porcine parvovirus

Porcine rotavirus (PoRV) belongs to rotavirus (RV), which is a genus in the Reoviridae family of double-stranded RNA (dsRNA) viruses [87]. PoRV is widely distributed worldwide, and epidemiological and experimental studies have confirmed that it has zoonotic transmission potential [88]. Since the end of 2010, large-scale outbreaks of severe diarrhea in piglets caused by PoRV have occurred in many parts of China. In a report from 2018, 65 out of 226 samples collected from suckling and weaned pigs from 10 farms in Shandong province with diarrhea were found to be positive for PoRV (28.76%) [89]. In a report from 2018 to 2019, a survey of porcine diarrhea viruses in Guangdong Province, China, revealed a detection rate of 18.6% for rotavirus [90].

RV primarily spreads through the fecal-oral route and replicates in mature, undivided enterocytes in the small intestine. Pancreatic proteases activate RV and promote virus entry into cells. The virus particularly affects the middle and tip of the villi, causing damage and eventually leading to villus atrophy [91].

The Parvoviridae family encompasses small, nonenveloped viruses with a linear, single-stranded DNA genome [92]. Parvovirus (PPV) infects a range of vertebrate hosts, including humans, pigs, dogs, cats, and birds [93]. Hemophilia patients were treated with porcine clotting factor VIII, in which PPV1 DNA was detected [94]. Furthermore, rodent H1 parvovirus has been demonstrated to be capable of replicating within human cells, posing the potential for pathogenicity in allograft environments [93].

PPV causes reproductive failure in pigs, characterized by embryonic and fetal death. The disease in a herd manifests with a decrease in litter size and an increase in the number of mummified and/or stillborn piglets [95]. PPV infection is endemic in most swine herds [96].

#### Newly emerged notable swine viruses

In addition to some porcine viruses that have been studied, newly emerging viruses may also cause failures in heterografts and need to be taken into consideration. Examples include severe acute respiratory syndrome coronavirus (SARS-CoV), porcine delta coronavirus (PDCoV), and Senecavirus A (SVA).

SARS-CoV is a type of coronavirus that was first identified in southern China in 2017, causing the death of approximately 24,500 piglets [97]. It re-emerged in pig herds in Guangdong in January 2019, with 13 out of 18 serologically tested healthy sows (72.2%) being SADS-CoV positive [98]. This virus primarily attacks the gastrointestinal system [99].

PDCoV is a new variant of coronaviruses that has been found to infect a wide range of species, including humans. This virus was discovered in pig herds in Hong Kong in the late 2000s and infects intestinal epithelia, causing watery diarrhea and vomiting. PDCoV enters cells via the aminopeptidase N (APN) receptor on the cell surface, which is widely distributed and highly conserved in nature, which may endow PDCoV with cross-species transmission abilities [99].

SVA is classified into the genus Senecavirus in the family Picornaviridae. Its related disease was not known until 2007 [100]. This virus can cause vesicular disease and epidemic transient neonatal losses in swine. It was discovered in pig farms in Guangdong Province, China, in 2015, causing vesicular lesions. By December 2019, more than half of the regions in China reported SVA infection impacts [101]. Notably, SVA is considered to infect human cells, but the pathways it mediates in human cells are different from those in pig cells [100].

### New pathways of virus transmission

#### Vertical transmission through somatic cell nuclear transfer (SCNT)

SCNT is the most common method for producing genetically edited pigs or transgenic pigs and allows for prescreening of cells. However, this method also carries the risk of infecting the animal with viruses, as it requires penetrating the protective zona pellucida (ZP) of the cell. The transmission ability of different viruses through the SCNT pathway is different. Some viruses can be infected by penetrating cell membranes during nuclear transfer, while some viruses (such as PREV and PCMV) may spread with the nucleus. For example, reproductive porcine viruses have an ability to infect embryos without zona pellucida, but PRRSV cannot infect early embryos because of the lack of viral receptors [102, 103]. PCV2 has been proven to spread via SCNT [104], and both PCV3 and PCMV are thought to be capable of spreading through SCNT in theory and have been confirmed in recent experiments [105].

In light of SCNT transmission, it is necessary to perform virus screening prior to the experiment and select animals that are virus-negative. Additionally, virus infection can be controlled through methods such as embryo washing and trypsin decontamination [104]. In addition to SCNT, expanding the production methods for genetically edited pigs, such as through the use of adeno-associated viral vectors for gene editing, is also a means to address this issue.

#### Interspecies transmission

In addition to the occurrence of transmission within pig populations, interspecies transmission is an issue of concern. Influenza A virus (IAV) represents a classic example of interspecies transmission, which results in the manifestation of swine influenza, a highly pathogenic pig disease. Currently, three primary subtypes of IAV, H1N1, H3N2, and H1N2, are prevalent globally within pig populations [106]. A reassortant Eurasian avian-like H1N1 virus, which emerged as dominant in pig populations since 2016, exhibits the ability to bind to human receptors, with a seroprevalence of over 10% among pig farm workers [107]. The virus can facilitate bidirectional transmission between pigs and humans, leading to the widespread outbreak of illness within pig populations.

In addition to humans, certain other animals may serve as hosts for the virus. For instance, all PRV virus can infect most non-primate mammals, such as goats, dogs, cats and wild animals such as opossums, raccoons. However, it is noteworthy that Clade 2 of PRV may be able to infect humans, which increases the risk of cross-infection of clade 2 of PRV among humans, pigs, and wild animals [108]. As a result, the detection of the virus in pig farm managers is imperative, and the management of pig farms should be tightened and strictly regulated to prevent cross-infection from other animals.

## Summary and prospects

Clinical surgery has indicated that virus safety is the key to the survival time of heterograft recipients. The presence of viruses in the donor pig that can cause disease in the recipient is a crucial issue to consider in clinical surgery. Therefore, not only should we carry out prevention and control measures, but we should also actively detect viruses and produce antiviral animals. However, new viruses and potential viruses continue to emerge, and the transmission of viruses is also a concern, but we have solutions for these issues as well. In the future, we hope to develop appropriate vaccines for these pathogens, monitor pathogens using next-generation sequencing (NGS), establish a swine population suitable for heterografts that are free of zoonotic diseases, and completely resolve the issue of microbiological safety in heterografts.

## Data Availability

Not applicable.
